# Discrepancy between official records of the Brazilian National Immunization Program Information System and the household survey about vaccination coverage in Cubatão, São Paulo: cross-sectional study, 2023

**DOI:** 10.1590/S2237-96222025v34e20240523.en

**Published:** 2025-08-11

**Authors:** Luciana Martins Rozman, Ana Marli Christovam Sartori, Alexandra Azevedo Souza, Patricia Coelho de Soarez

**Affiliations:** 1 Universidade São Judas Tadeu, Cubatão, SP, Brasil; 2 Universidade de São Paulo, Faculdade de Medicina, São Paulo, SP, Brazil; 3 Secretaria Municipal de Saúde de Cubatão, Cubatão, SP, Brazil; 4 Universidade de São Paulo, Faculdade de Medicina, Departamento de Medicina Preventiva, São Paulo, SP, Brazil

**Keywords:** Information Systems, Immunization Programs, Vaccination Coverage, Epidemiological Survey, Cross-Sectional Studies, Sistemas de Información, Programas de Inmunización, Cobertura de Vacunación, Encuestas Epidemiológicas, Estudios Transversales

## Abstract

**Objective:**

To assess the concordance between vaccination data registered in the Brazilian National Immunization Program Information System and information in the vaccination booklets of children under 2 years of age who participated in the household survey of vaccination coverage in Cubatão, São Paulo.

**Methods:**

This is a descriptive study comparing the doses registered in vaccination booklets with those in the Brazilian National Immunization Program Information System for children born in 2019 and 2020. Concordance was assessed regarding the doses applied and application dates recorded, using the Kappa test and the intraclass correlation coefficient.

**Results:**

The household survey showed that vaccination coverage targets were achieved for all vaccines in the national immunization schedule for children under 2 years of age: 90.0% for BCG and human rotavirus, and 95.0% for other vaccines. When checking the same children in the information system, coverage was below 70.0%, and in 10.0% of them the application dates were inconsistent. Approximately 24.0% of the children were not entered in the information system, despite having their vaccinations recorded in their booklets.

**Conclusion:**

The results indicate issues regarding accuracy and completeness of data from the Brazilian National Immunization Program Information System compared to vaccination booklets, highlighting challenges in vaccination records that may compromise monitoring of vaccination coverage in Cubatão.

## Resumen

**Objetivo:**

Evaluar la concordancia entre los datos de vacunación registrados en el Sistema de Información del Programa Nacional de Inmunizaciones y la información en los carnés de vacunación de niños menores de 2 años que participaron en la encuesta domiciliaria de cobertura vacunal en Cubatão, São Paulo.

**Métodos:**

Se trata de un estudio descriptivo que compara las dosis registradas en los carnés de vacunación con las del Sistema de Información del Programa Nacional de Inmunizaciones para niños nacidos en 2019 y 2020. La concordancia se evaluó en cuanto al registro de las dosis aplicadas y las fechas de aplicación, utilizando la prueba de Kappa y el coeficiente de correlación intraclase.

**Resultados:**

La encuesta domiciliaria mostró que se alcanzaron las metas de cobertura vacunal para todas las vacunas del calendario nacional de inmunización para menores de 2 años: 90,0% para BCG y rotavirus humano, y 95,0% para las demás vacunas. Al verificar a los mismos niños en el sistema de información, la cobertura fue inferior al 70,0%, y en el 10,0% de ellos las fechas de aplicación eran discordantes. Aproximadamente el 24,0% de los niños no tenían registro en el sistema de información, a pesar de tener las vacunas registradas en los carnés.

**Conclusión:**

Los resultados indican problemas en la precisión y completitud de los datos del Sistema de Información del Programa Nacional de Inmunizaciones en comparación con los carnés de vacunación, evidenciando desafíos en el registro de vacunación que pueden comprometer el monitoreo de las coberturas vacunales en Cubatão.

## Introduction

Routine vaccination coverage in the Brazilian National Vaccination Calendar of the Brazilian National Immunization Program has fallen in recent years (1,2) and in 2019 no vaccine in the childhood vaccination calendar reached the recommended target (3).

Factors such as the complexity of the vaccination schedule, the temporary shortage of immunobiologicals, the lack of professional training, the restricted opening hours of vaccination spaces and the underfunding of Primary Care may explain this decrease (4). Lack of knowledge about the importance of vaccination, the false impression generated by the reduction in vaccine-preventable diseases, hesitancy to vaccinate, fake news on social media and health inequalities are also mentioned in the literature (5). The covid-19 pandemic further worsened the situation, reducing vaccination coverage by 20.0% (6).

Managers of the Brazilian Unified Health System (Sistema Único de Saúde, SUS) argue that the drop in vaccination coverage may be related to changes in the vaccination data registration system in the country (7) and the delay in transferring data sent by municipal health departments to the national database, with reports of loss of information during the process and delay in data transfer (8).

The implementation of the Brazilian National Immunization Program Information System began in 2010, enabling the individual registration of each vaccinated person with identification of their place of residence, monitoring of their vaccination status and scheduling of vaccines as provided for in the national calendar (9,10). 

Despite the benefits of the Brazilian National Immunization Program Information System and incentives from the Ministry of Health (11,12), its implementation was slow and heterogeneous among municipalities. In 2016, the implementation process in municipalities was below 60.0% (13). In July 2019, 36,070 active vaccination spaces were registered, and of these, 28,874 (80.0%) adopted the use of the system (3).

In the second half of 2019, the system underwent reformulation with the aim of modernizing the platforms and functionalities, making the digital vaccination booklet available and meeting the new e-government strategies of the National Health Information and Informatics Policy, combined with the World Health Organization guidelines for the Electronic Vaccination Registry (3). With the aim of enabling a single data entry for Primary Care services, the Ministry of Health promoted the integration of the vaccination databases of the Primary Care Information System with the Brazilian National Immunization Program Information System (14). 

The doses administered in basic health units began to be recorded in e-SUS Basic Care, and the Brazilian National Immunization Program Information System continued to be used by special immunobiological reference centers, private vaccination clinics, hospitals and maternity wards (15). The data entered in vaccination rooms of the basic health units are sent to the national database of the Brazilian National Immunization Program Information System through the health interoperability platform, called the National Health Data Network (16). After a validation process that includes identifying the user through the Individual Taxpayer Registry or the National Health Card, data from basic health units are added to data from other types of health establishments and are available for extraction for vaccination monitoring reports (17). 

The Brazilian National Immunization Program Information System continues to be the main instrument for estimating vaccination coverage and guiding vaccination actions(3). Few studies have evaluated its effectiveness in estimating coverage of vaccines in the national childhood calendar (8,18). Vaccination coverage surveys can help validate this system, by comparing it with data from vaccination records. The municipality of Cubatão carried out a household survey on the vaccination coverage of children under 2 years old, born in 2019 and 2020, generating data that help discussing the impact of data quality on the information system.

The objective of this study was to evaluate the concordance between vaccination data recorded in the Brazilian National Immunization Program Information System and the information found in the vaccination booklets of children under 2 years of age who participated in the vaccination coverage household survey in Cubatão. 

## Methods

### Study design

This is a cross-sectional study that compared primary and secondary data on vaccination coverage of children under 2 years of age born in the period 2019-2020 in Cubatão, São Paulo. Primary data were obtained through a household survey carried out between March and July 2023. Secondary data were extracted from the Brazilian National Immunization Program Information System in August 2023.

### Context

The study was carried out in Cubatão, located on the coast of the state of São Paulo, with 112,476 inhabitants and around 1,500 live births per year. The Primary Care network has 18 basic health units, 4 of which do not have a vaccination room. Primary Care coverage in December 2023 was 43.9% (58,154 people) (19).

### Participants

The survey population was made up of children born alive in the period 2019-2020 (n=2,950), residing in Cubatão who, in March 2023, would be between 39 and 50 months old. Population data were obtained from the Live Birth Information System.

### Variables

The variables analyzed include the vaccine doses that make up the national calendar for children under 2 years of age: BCG, hepatitis B at birth, pentavalent, inactivated polio vaccine, pneumococcal conjugate, meningococcal conjugate, rotavirus, yellow fever, measles, mumps and rubella, chickenpox, hepatitis A, first polio booster and first diphtheria, tetanus and pertussis booster. 

### Data sources/measurement

To collect data from the household survey, the municipality was divided into socioeconomic strata, considering the population’s living conditions. For this division, health professionals from the municipality with experience in the territory were consulted. 

The addresses of children born alive in 2019 and 2020 were georeferenced, according to data from the Live Birth Information System. Then, clusters of census sectors with 28 or more live births were formed for each stratum. A conglomerate could be formed by one or more census sectors as long as they were geographically contiguous and socioeconomically homogeneous. In each of the strata, the clusters to be included in the sample were drawn by means of systematic sampling. Data collection from home interviews was carried out using a standardized questionnaire. During the interviews, the vaccination booklets were photographed so that the data on doses administered could later be read and entered by healthcare professionals with experience in the area. 

The doses recorded in the booklets were compared with the doses recorded in the Brazilian National Immunization Program Information System, to assess the concordance in the registration of the dose applied and the concordance in terms of dates of application. 

### Study size

The parameters used to calculate the survey sample size were: expected proportion of vaccinated children equal to 0.70, significance level of 0.05 and design effect equal to 1.50. Based on these values, the sample size was estimated at 450 children, and each stratum was composed of 90 live births.

### Statistical methods

The concordance between the records in the booklets and the records in the Brazilian National Immunization Program Information System was assessed using the Kappa test for categorical variables (vaccinated, unvaccinated), categorized as follows: perfect concordance when the Kappa coefficient was equal to 1; almost perfect concordance, ≤0.80-<1; substantial concordance, 0.60-0.79; moderate concordance, 0.41-0.59; fair concordance, 0.21-0.40; poor concordance, ≤0-<0.20; and no concordance when the value was <0. 

The Kappa test is a reliability quantitative measure for two sources of information, corrected for the frequency with which the sources might agree by chance. Considering the importance of reliability in immunization information systems for decision-making, concordance below 0.80 was seen as inadequate (20).

For those with a record of the dose applied in both sources, the level of concordance between the dates of vaccine application was assessed, using the intraclass correlation coefficient. This coefficient also varies from 0 to 1 and followed the same categorization as the Kappa test. In both tests, a significance level of 5.0% was adopted and, for data analysis, the R software, version 4 4.2, was used.

## Results

407 families completed the household survey, which represents 90.4% of the expected sample. Missing respondents were due to refusal to complete the questionnaire (n=18), the impossibility of carrying out the interview after two visits at different times (n=3) and the difficulty in finding the expected number of children, even after an active search. Whenever possible, refusals were replaced by other children from the conglomerate.

Of the 407 participating children, 311 had data recorded in the Brazilian National Immunization Program Information System, meaning that 23.6% were not registered in the system, although they had a vaccination booklet.

**Figure 1:**
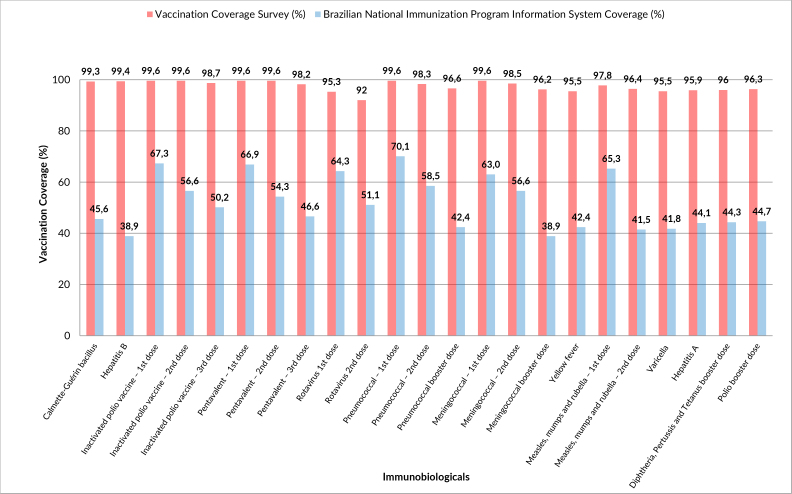
Proportion of vaccination coverage, according to data source: vaccination coverage survey and Brazilian National Immunization Program Information System. Cubatão, 2023 (n=407)

The coverage observed in the survey (vaccination booklet) reached the recommended targets (90.0% for BCG and human rotavirus and 95.0% for the other vaccines) and was higher than that found in the Brazilian National Immunization Program Information System for all vaccines (Figure 1). 

Concordance was observed between the vaccination recorded in the booklets and in the Brazilian National Immunization Program Information System for the 23 vaccines that are part of the national immunization schedule for children under 2 years of age (Table 1). The vaccine with the lowest proportion of records in the Information System was hepatitis B. Of the 311 children found in the information system, 60.8% had no record of this vaccine, with this dose generally being administered in maternity wards shortly after birth. For all vaccines analyzed, the concordance between the two sources of information was poor (Kappa≤0-<0.20) or non-existent (Kappa<0). This result was statistically significant for the following vaccine doses: first dose of human rotavirus (Kappa 0.113: p-value<0.001), second dose of rotavirus (Kappa 0.114; p-value<0.001), second dose of meningococcal (Kappa 0.033; p-value 0.035) and first dose of measles, mumps and rubella (Kappa 0.056; p-value 0.018) vaccines (Table 1). 

**Table 1:** Number of children with doses registered in the vaccination booklet and in the Brazilian National Immunization Program Information System and Kappa concordance between the two sources of information. Cubatão, 2023 (n=407)

Immunobiologicals	vaccination booklets (n=407)	No registration in the information system n=311 (%)	Kappa coefficient	p-value
BCG	404	167 (53.7)	0.011	0.502
Hepatitis B	404	188 (60.8)	-0.002	1.000
Inactivated polio vaccine – 1^st^ dose	406	102 (32.8)	-0.006	1.000
Inactivated polio vaccine – 2^nd^ dose	406	134 (43.1)	0.008	0.434
Inactivated polio vaccine – 3^rd^ dose	402	151 (48.6)	0.026	0.061
Pentavalent – 1^st^ dose	406	102 (33.1)	-0.006	1.000
Pentavalent – 2^nd^ dose	406	141 (45.3)	0.008	0.457
Pentavalent – 3^rd^ dose	400	161 (51.8)	0.028	0.063
Human rotavirus – 1^st^ dose	394	101 (32.5)	0.113	<0.001
Human Rotavirus – 2^nd^ dose	385	135 (43.4)	0.114	<0.001
Pneumococcal – 1^st^ dose	406	92 (29.9)	-0.006	1.000
Pneumococcal – 2^nd^ dose	406	128 (41.2)	0.009	0.415
Pneumococcal – booster	398	172 (55.6)	0.023	0.245
Meningococcal – 1^st^ dose	406	114 (37.0)	-0.006	1.000
Meningococcal – 2^nd^ dose	402	131 (42.1)	0.033	0.035
Meningococcal – booster	398	183 (59.2)	0.019	0.252
Yellow fever	386	163 (54.0)	0.024	0.316
Measles, mumps, and rubella – 1^st^ dose	399	102 (32.5)	0.056	0.018
Measles, mumps, and rubella – 2^nd^ dose	391	170 (55.6)	0.024	0.249
Varicella	391	168 (54.7)	0.035	0.078
Hepatitis A	391	162 (52.7)	0.039	0.072
Diphtheria, tetanus, and whooping cough – 1^st^ booster	393	159 (51.8)	0.037	0.070
Polio – 1^st^ booster	397	164 (53.4)	0.019	0.300

The concordance of vaccine application dates was evaluated comparing the cases recorded in the booklet and in the information system. Yellow fever and first booster doses of polio and diphtheria, tetanus and pertussis vaccines showed a degree of concordance between 0.60 and 0.79 (substantial concordance). All others showed concordance above 0.80 (almost perfect concordance). It is noteworthy that vaccines that must be administered on the same date presented different concordance proportions, for example, the BCG (90.2%) and hepatitis B (92.6%) vaccines, the first booster doses of diphtheria, tetanus and whooping cough (72.3%) and poliomyelitis (77.7%) and the second dose of measles, mumps and rubella (72.1%) and chickenpox (90.0%). The difference in registration of application dates in the information system ranged from 1 day (hepatitis A) to -1,186 days (yellow fever) in relation to the vaccination booklet (Table 2).

**Table 2:** Number of children with registered vaccination in the vaccination booklet and in the Brazilian National Immunization Program Information System, number of children with registered application dates concordant in both data sources, intraclass correlation coefficient (intraclass correlation coefficient – ICC) and difference in days of cases with discordant dates by immunobiological. Cubatão, 2023 (n=311)

	Number of cases				Minimum and maximum values when there is mismatch between dates (days)	
Immunobiological	With vaccine registration in the Information System (n=311)	With concordance in application date n=311 (%)	ICC	p-value of ICC	Minimum	Maximum
BCG	142	128 (90.2)	0.907	<0.001	-1095	335
Hepatitis B	121	112 (92.6)	0.997	<0.001	-78	120
Inactivated polio vaccine – 1^st^ dose	209	191 (91.4)	0.998	<0.001	-98	3
Inactivated polio vaccine – 2^nd^ dose	176	152 (86.4)	0.925	<0.001	-833	365
Inactivated polio vaccine – 3^rd^ dose	156	138 (88.4)	0.950	<0.001	-850	188
Pentavalent – 1^st^ dose	208	191 (91.8)	0.998	<0.001	-112	2
Pentavalent – 2^nd^ dose	169	146 (86.4)	0.930	<0.001	-833	61
Pentavalent – 3^rd^ dose	145	126 (86.9)	0.944	<0.001	-850	188
Human rotavirus – 1^st^ dose	200	186 (93.0)	0.989	<0.001	-77	366
Human Rotavirus – 2^nd^ dose	159	143 (89.9)	0.932	<0.001	-833	366
Pneumococcal – 1^st^ dose	218	198 (90.8)	0.963	<0.001	-440	31
Pneumococcal – 2^nd^ dose	182	161 (88.5)	0.919	<0.001	-833	132
Pneumococcal – booster	132	113 (85.6)	0.957	<0.001	-662	366
Meningococcal – 1^st^ dose	196	157 (80.1)	0.926	<0.001	-436	89
Meningococcal – 2^nd^ dose	176	138 (78.4)	0.895	<0.001	-792	101
Meningococcal – booster	121	111 (91.7)	0.970	<0.001	-524	217
Yellow fever	132	113 (85.6)	0.731	<0.001	-1186	1181
Measles, mumps and rubella – 1^st^ dose	203	110 (54.2)	0.809	<0.001	-693	784
Measles, mumps and rubella – 2^nd^ dose	129	93 (72.1)	0.870	<0.001	-512	731
Varicella	130	117 (90.0)	0.875	<0.001	-1044	425
Hepatitis A	137	130 (94.9)	0.989	<0.001	-365	1
Diphtheria, tetanus and whooping cough – 1^st^ booster	141	102 (72.3)	0.770	<0.001	-961	716
Polio – 1^st^ booster	139	108 (77.7)	0.710	<0.001	-1044	478

## Discussion

This study revealed discrepancies between vaccination dose records in the booklets and data from the Brazilian National Immunization Program Information System. Although the results from vaccination booklets show that Cubatão reached the vaccination coverage targets for children under 2 years old born in 2019 and 2020, the low completeness and accuracy of the data in the Brazilian National Immunization Program Information System highlight challenges in recording and managing information. Such discrepancies may compromise the monitoring of vaccination coverage, affecting the ability to assess the effectiveness of immunization strategies. These findings reinforce the need to improve the quality of records, to ensure that vaccination efforts are adequately documented and evaluated.

Good quality vaccination records are essential for monitoring and evaluating vaccination coverage and immunization programs (21). An effective information system for monitoring vaccination coverage at different levels of health care should include essential components, such as: reliable internet access in vaccination rooms, interoperability with other databases, such as live birth registration, sufficient and well-trained human resources, adequate funding, procedures for dealing with duplicate records and unique identification in the system, as well as procedures to prevent data loss and ensure rapid feedback between national and local levels (22). To be considered of good quality, records must be accurate, complete, relevant and timely (23). Poor quality vaccination records have been an obstacle for low- and middle-income countries (24,25). 

There are multiple challenges to implementing a national nominal vaccination registration system in a country of continental dimensions and with great regional and socioeconomic inequality, such as Brazil. Among the challenges are the lack of equipment, poor internet connection and lack of professionals trained in services located in more remote areas. One notable obstacle is the existence of older registration systems developed by municipalities with more resources, which do not want to stop using their own system. 

The loss of information during the process of data transmission from municipalities to the Brazilian National Immunization Program Information System is mentioned as another challenge for the information system. Comparing the records for diphtheria, tetanus and whooping cough from the vaccination rooms of a municipality in Minas Gerais with those transferred to the national database of the National Immunization Program, concordance was found for only 1 of the 60 (1.6%) months analyzed (8).

The migration of the information registration system in 2019, which then began register the doses applied in health units in e-SUS Primary Care before the complete implementation of the Brazilian National Immunization Program Information System (3), brought difficulties related to the complexity of implementing the new system in the vaccination rooms and ensuring that the doses were correctly recorded and transferred to the central level without losses.

e-SUS Primary Care is a system that aims to simplify the collection, insertion, management and use of information at the Primary Care level, playing a vital role in the SUS information network (26). The system consists of two separate software programs, as described below.

1. Simplified Data Collection: transition/contingency system that supports the data collection process through forms for recording information on actions carried out by Primary Care teams. It is recommended for healthcare establishments that do not have an internet connection or enough computers for its professionals.
2. Electronic Citizen Record: a system with electronic records whose main objective is to support the computerization process of basic health units. It is used to enter clinical records of care and Simplified Data Collection system forms, filled out by the team in their work process (27).

In Cubatão, although health units had computers and internet connection, the information system used by Primary Care until the beginning of 2024 was e-SUS Primary Care – Simplified Data Collection, which allows the individual entry of vaccine doses applied. Unlike the Electronic Citizen Record, the identification field with data from the National Health Card or the Individual Taxpayer Registry is not mandatory (28), and the absence of this data makes interoperability between systems impossible. 

An assessment by the Municipal Health Department of Cubatão, carried out between January and June 2023, found out that the number of data recorded manually in vaccination rooms was 23.4% higher than that observed in the municipal e-SUS Primary Care reports and 58.0% higher than the data from the Brazilian National Immunization Program Information System (unpublished data). 

The Ministry of Health has focused its efforts on recovering data by identifying withheld doses that do not reach the National Health Data Network. In 2023, 91.0% of the doses held back in the Primary Care information system were attributed to the error “patient not found”, making them ineligible for immediate submission to the National Health Data Network. Among these doses, 99.6% were held back due to wrong or missing identification number (National Health Card or Individual Taxpayer Registry). In order to correct this error, municipalities have received a spreadsheet detailing all doses held back for this reason, so that the necessary corrections can be made and the data can be resubmitted (28). 

In addition to the discrepancy between data from the Brazilian National Immunization Program Information System and data recorded in the Cubatão, São Paulo vaccination booklets, this study revealed that 23.6% of the children included in the vaccination survey in this municipality did not have any record in the information system and in 10% of cases there was also discrepancy in the dates of vaccination. 

Among 4,050 children with a completed vaccination scheme at 24 months, 11.0% had no record in the Brazilian National Immunization Program Information System, with the total discrepancy between doses and dates in the two sources being 14.0% (18). The higher percentage of missing records in this study may be due to the methodology used. To assess the reliability of the information system, the subsample of children included in the survey who had a complete vaccination scheme was used. In this study, the entire sample was evaluated, regardless of the vaccine status. In a province in China, it was revealed that the proportion of children with vaccination records and no registration in the information system was 12.6%, also lower than that of this study (29). 

One of the possible explanations for these discrepancies is the practice of not typing the data directly into the system at the time of vaccination. The use of paper as a means of recording and consequently not typing in real time can generate under-recording and compromise data quality. Factors such as poor internet connectivity, slow system performance and the fact that vaccination data is sometimes entered by another professional are highlighted as impediments to real-time entry (13,28,30,31). Another factor that may have contributed to the lack of registration during this period was the working conditions imposed by the Covid-19 pandemic. Due to the increase in cases of illness among professionals and the resizing of teams in hospitals and emergency care units, it was necessary to reallocate workers to frontline sectors to care for COVID-19 cases, overloading other areas (32).

A critical issue for improving data at the municipal level is the implementation of a “culture of using data”. There is an understanding that without the continuous use of the information generated, there will be no incentive to improve data collection (33). Low-quality data will not be used and, precisely because it is not used, its quality will remain low. The expanded use of data contributes to improving data collection and data quality, which promotes greater use of data (34). 

Training and encouraging municipalities to use data for decision-making can be a strategy for the constant monitoring and early identification of failures in data collection. Supervision of vaccination rooms and feedbacks from central levels have also been highlighted as strategies for improving quality (35), bringing an element of shared responsibility in relation to data quality (34).

International experiences point to cell phones, already widely used in Brazil, as a tool to register the doses applied through applications in a more agile and timely way, even in contexts of poor connectivity. Successful examples in countries such as Mozambique, China and Thailand reinforce the viability of these solutions in scenarios with limited resources (36).

The implementation of technology, such as an immunization information system, involves structural and procedural changes in the daily lives of health professionals and constitutes a complex process (30). The results of this study highlight challenges in vaccination registration in Cubatão, especially regarding the accuracy and completeness of data in the Brazilian National Immunization Program Information System. The discrepancies between the data registered in the booklets and in the information system can be attributed, in part, to the lack of real-time typing and the absence of correct patient identification records, compromising the interoperability of the e-SUS and the Brazilian National Immunization Program Information System.

It is essential to intensify efforts to train health teams, improve technological infrastructure and ensure accurate and timely recording of vaccination data. Recovering withheld doses and correcting incorrect records are essential to improve the quality of information and, consequently, the efficiency of immunization programs. Future studies should continue to monitor vaccination coverage and evaluate the impact of interventions implemented, ensuring that vaccination targets are met and accurately reflected in official records.

## Data Availability

Following ethical and legal guidelines, and to ensure the protection of participants’ privacy and compliance with the rules of the relevant ethics committee, the data generated in this research will be available upon request.
