# Supplementary material for: Discrepancy between official records of the Brazilian National Immunization Program Information System and the household survey about vaccination coverage in Cubatão, São Paulo: cross-sectional study, 2023
Source: Epidemiol Serv Saude. 2025 Aug 11;34:e20240523. doi: 10.1590/S2237-96222025v34e20240523.en (PMC12342853; doi:10.1590/S2237-96222025v34e20240523.en)
Supplement: Translation

# Translation: Descompasso entre registros oficiais do Sistema de Informações do Programa Nacional de Imunizações e inquérito domiciliar de cobertura vacinal em Cubatão, São Paulo: estudo transversal, 2023

**DOI:** 10.1590/S2237-96222025v34e20240523.pt

**Authors:** Luciana Martins Rozman, Ana Marli Christovam Sartori, Alexandra Azevedo Souza, Patricia Coelho de Soarez

**Affiliations:** 1 Universidade São Judas Tadeu, Cubatão, SP, Brasil; 2 Universidade de São Paulo, Faculdade de Medicina, São Paulo, SP, Brasil; 3 Secretaria Municipal de Saúde de Cubatão, Cubatão, SP, Brasil; 4 Universidade de São Paulo, Faculdade de Medicina, Departamento de Medicina Preventiva, São Paulo, SP, Brasil

**Keywords:** Sistemas de Informação, Programas de Imunização, Cobertura Vacinal, Inquérito Epidemiológico, Estudos Transversais

## Abstract

**Objetivo:**

Avaliar a concordância entre os dados de vacinação registrados no Sistema de Informações do Programa Nacional de Imunizações e as informações nas cadernetas de vacinação de crianças menores de 2 anos que participaram do inquérito domiciliar de cobertura vacinal em Cubatão, São Paulo.

**Métodos:**

Trata-se de estudo descritivo comparando as doses registradas nas cadernetas de vacinação com as do Sistema de Informações do Programa Nacional de Imunizações para crianças nascidas em 2019 e 2020. A concordância foi avaliada quanto ao registro das doses aplicadas e das datas de aplicação, utilizando o teste de Kappa e o coeficiente de correlação intraclasse.

**Resultados:**

O inquérito domiciliar mostrou que as metas de cobertura vacinal foram atingidas para todas as vacinas do calendário nacional de imunização para menores de 2 anos: 90,0% para BCG e rotavírus humano, e 95,0% para as demais vacinas. Ao verificar as mesmas crianças no sistema de informações, a cobertura ficou abaixo de 70,0%, e em 10,0% delas as datas de aplicação eram discordantes. Cerca de 24,0% das crianças não possuíam registro no sistema de informações, apesar de terem as vacinas registradas nas cadernetas.

**Conclusão:**

Os resultados indicam problemas na precisão e completude dos dados do Sistema de Informações do Programa Nacional de Imunizações em comparação com as cadernetas de vacinação, evidenciando desafios no registro de vacinação que podem comprometer o monitoramento das coberturas vacinais em Cubatão.

## Introdução

As coberturas vacinais de rotina do Calendário Nacional de Vacinação do Programa Nacional de Imunizações apresentaram queda nos últimos anos (1,2) e, em 2019, nenhuma vacina do calendário infantil atingiu a meta preconizada (3).

Fatores como a complexidade do calendário vacinal, o desabastecimento temporário de imunobiológicos, a falta de capacitação dos profissionais, o horário restrito das salas de vacina e o subfinanciamento da Atenção Básica podem explicar essa diminuição (4). O desconhecimento sobre a importância da vacinação, a falsa impressão gerada pela redução das doenças imunopreveníveis, a hesitação em vacinar, as falsas notícias nas redes sociais e as desigualdades em saúde também são mencionados na literatura (5). A pandemia de covid-19 agravou ainda mais a situação, reduzindo as coberturas vacinais em 20,0% (6).

Gestores do Sistema Único de Saúde (SUS) argumentam que a queda da cobertura vacinal pode estar relacionada às mudanças no sistema de registro de dados de vacinação no país (7) e à demora na transferência dos dados enviados pelas secretarias municipais de saúde para base de dados nacional, com relatos de perda de informação durante o processo e atraso no repasse de dados (8).

O Sistema de Informações do Programa Nacional de Imunizações teve sua implantação iniciada em 2010, possibilitando o registro individual de cada vacinado com identificação do local de residência, o acompanhamento da situação vacinal e o aprazamento das vacinas previstas no calendário nacional (9,10). 

Apesar dos benefícios do Sistema de Informações do Programa Nacional de Imunizações e dos incentivos do Ministério da Saúde (11,12), sua implantação ocorreu de forma lenta e heterogênea entre os municípios. Em 2016, o processo de implantação nos municípios encontrava-se com percentuais abaixo de 60,0% (13).

Em julho de 2019, foram registradas 36.070 salas ativas, e, destas, 28.874 (80,0%) aderiram ao uso do sistema (3).

No segundo semestre de 2019, o sistema passou por reformulação com a finalidade de modernizar as plataformas e as funcionalidades, disponibilizar a caderneta eletrônica de vacinação e atender às novas estratégias de governo eletrônico, da Política Nacional de Informação e Informática em Saúde, combinadas com as orientações da Organização Mundial da Saúde para o Registro Nominal de Vacinação Eletrônico (3). Com o objetivo de possibilitar uma única entrada de dados para os serviços da Atenção Básica, o Ministério da Saúde promoveu a integração das bases de dados de vacinação do Sistema de Informação da Atenção Básica com o Sistema de Informações do Programa Nacional de Imunizações (14). 

As doses aplicadas nas unidades básicas de saúde passaram a ser registradas no e-SUS Atenção Básica, e o Sistema de Informações do Programa Nacional de Imunizações continuou a ser usado pelos centros de referência de imunobiológicos especiais, clínicas de vacinação privadas, hospitais e maternidades (15). Os dados digitados nas salas de vacina das unidades básicas de saúde são enviados à base nacional do Sistema de Informações do Programa Nacional de Imunizações por meio da plataforma de interoperabilidade em saúde denominada Rede Nacional de Dados em Saúde (16). Após processo de validação que compreende a identificação do usuário pelo Cadastro de Pessoas Físicas ou pelo Cartão Nacional de Saúde, os dados das unidades básicas de saúde são somados aos dados dos demais tipos de estabelecimentos de saúde e ficam disponíveis para extração para os relatórios de monitoramento da vacinação (17). 

O Sistema de Informações do Programa Nacional de Imunizações continua sendo o principal instrumento para estimar coberturas vacinais e orientar as ações de vacinação (3). Poucos estudos avaliaram sua eficácia para estimar a cobertura das vacinas do calendário nacional infantil (8,18). Inquéritos de cobertura vacinal podem auxiliar na validação desse sistema comparando com os dados das cadernetas. O município de Cubatão realizou inquérito domiciliar sobre a cobertura vacinal de crianças menores de 2 anos, nascidas em 2019 e 2020, cujos dados ajudam a discutir o impacto da qualidade dos dados no sistema de informações.

O objetivo deste estudo foi avaliar a concordância entre os dados de vacinação registrados no Sistema de Informações do Programa Nacional de Imunizações e as informações encontradas nas cadernetas de vacinação de crianças menores de 2 anos que participaram do inquérito domiciliar de cobertura vacinal em Cubatão. 

## Métodos

### Delineamento do estudo

Trata-se de estudo transversal que comparou dados primários e secundários sobre a cobertura vacinal de crianças menores de 2 anos nascidas no período 2019-2020 em Cubatão, São Paulo. Os dados primários foram obtidos por meio de inquérito domiciliar realizado entre março e julho de 2023. Os dados secundários foram extraídos do Sistema de Informações do Programa Nacional de Imunizações em agosto de 2023.

### Contexto

O estudo foi realizado em Cubatão, localizado no litoral do estado de São Paulo, com 112.476 habitantes e cerca de 1,5 mil nascidos vivos por ano. A rede de Atenção Básica conta com 18 unidades básicas de saúde, sendo 4 unidades sem sala de vacinação. A cobertura de Atenção Básica em dezembro de 2023 era 43,9% (58.154 pessoas) (19).

### Participantes

A população do inquérito foi formada pelas crianças nascidas vivas no período 2019-2020 (n=2.950), residentes em Cubatão que, em março de 2023, teriam entre 39 e 50 meses de idade. Os dados populacionais foram obtidos do Sistema de Informação sobre Nascidos Vivos.

### Variáveis

As variáveis analisadas incluem as doses de vacinas que compõem o calendário nacional para menores de 2 anos de idade: BCG, hepatite B ao nascer, pentavalente, vacina inativada da poliomielite, pneumocócica conjugada, meningocócica conjugada, rotavírus, febre amarela, sarampo, caxumba e rubéola, varicela, hepatite A, primeiro reforço de poliomielite e primeiro reforço de difteria, tétano e coqueluche. 

### Fontes de dados/mensuração

Para a coleta de dados do inquérito domiciliar, o município foi dividido em estratos socioeconômicos, considerando-se as condições de vida da população. Para essa divisão, foram consultados profissionais de saúde do município com experiência no território. 

Realizou-se o georreferenciamento do endereço das crianças nascidas vivas em 2019 e 2020, de acordo com os dados do Sistema de Informação sobre Nascidos Vivos. Em seguida, foram formados, em cada estrato, conglomerados de setores censitários com 28 ou mais nascidos vivos. Um conglomerado poderia ser formado por um ou mais setores censitários desde que contíguos geograficamente e homogêneos socioeconomicamente. Em cada um dos estratos, foram sorteados, por meio de amostragem sistemática, os conglomerados a serem incluídos na amostra. A coleta de dados das entrevistas domiciliares foi realizada por meio de questionário padronizado. Durante as entrevistas, as cadernetas de vacinação foram fotografadas, para que os dados de doses aplicadas fossem posteriormente lidos e digitados por profissionais de saúde com experiência na área. 

As doses registradas nas cadernetas foram comparadas com as doses registradas no Sistema de Informações do Programa Nacional de Imunizações, para avaliar a concordância de registro de dose aplicada e concordância de datas de aplicação. 

### Tamanho do estudo

Os parâmetros utilizados para o cálculo do tamanho da amostra do inquérito foram: proporção esperada de crianças vacinadas igual a 0,70, nível de significância de 0,05 e efeito do desenho igual a 1,50. Com base nesses valores, estimou-se o tamanho da amostra em 450 crianças, e cada estrato foi composto por 90 nascidos vivos.

### Métodos estatísticos

A concordância entre os registros das cadernetas e os registros do Sistema de Informação do Programa Nacional de Imunizações foi avaliada utilizando o teste de Kappa para variáveis categóricas (vacinado, não vacinado), categorizado da seguinte forma: concordância perfeita quando o coeficiente de Kappa foi igual a 1; concordância quase perfeita, ≤0,80-<1; concordância substancial, 0,60-0,79; concordância moderada, 0,41-0,59; concordância regular, 0,21-0,40; concordância ruim, ≤0-<0,20; e nenhuma concordância quando o valor foi <0. 

O teste de Kappa é uma medida quantitativa de confiabilidade para duas fontes de informação, corrigida pela frequência com que as fontes podem concordar por acaso. Considerando a importância da confiabilidade nos sistemas de informação de imunização para tomadas de decisão, foi inadequada a concordância abaixo de 0,80 (20).

Para aqueles com registro de dose aplicada nas duas fontes, foi avaliado o nível de concordância entre as datas de aplicação da vacina, utilizando o coeficiente de correlação intraclasse. Esse coeficiente também varia de 0 a 1 e seguiu a mesma categorização do teste de Kappa. Em ambos os testes, adotou-se o nível de significância de 5,0% e, para a análise dos dados, foi utilizado o programa R, versão 4 4.2.

## Resultados

O inquérito domiciliar entrevistou 407 famílias, o que representa 90,4% da amostra prevista. As perdas ocorreram pela recusa (n=18), pela impossibilidade de realizar a entrevista após duas visitas em horários diferentes (n=3) e pela dificuldade de encontrar o número esperado de crianças, mesmo após busca ativa. As recusas eram substituídas, quando possível, por outra criança do conglomerado.

Das 407 crianças participantes, 311 tinham dados registrados no Sistema de Informações do Programa Nacional de Imunizações, ou seja, 23,6% não possuíam registro no sistema, embora tivessem caderneta de vacinação.

As coberturas observadas no inquérito (caderneta de vacinação) alcançaram as metas preconizadas (90,0% para BCG e rotavírus humano e 95,0% para as demais) e foram superiores às encontradas no Sistema de Informações do Programa Nacional de Imunizações para todas as vacinas (Figura 1). 

**Figura 1:** Proporção de cobertura vacinal, segundo fonte de dados: inquérito de cobertura vacinal e Sistema de Informações do Programa Nacional de Imunizações. Cubatão, 2023 (n=407)

Houve concordância entre os registros de vacinação nas cadernetas e no Sistema de Informações do Programa Nacional de Imunizações para as 23 vacinas que fazem parte do calendário nacional de imunização de menores de 2 anos (Tabela 1). A vacina com menor proporção de registros no Sistema de Informações foi a hepatite B. Das 311 crianças encontradas no sistema de informação, 60,8% não tinham registro dessa vacina, sendo essa dose, geralmente, administrada nas maternidades logo após o nascimento. Para todas as vacinas analisadas, a concordância entre as duas fontes de informação foi ruim (Kappa≤0-<0,20) ou inexistente (Kappa<0). Esse resultado foi estatisticamente significativo para as seguintes doses de vacina: primeira dose de rotavírus humano (Kappa 0,113: p-valor<0,001), segunda dose de rotavírus (Kappa 0,114; p-valor<0,001), segunda dose de meningocócica (Kappa 0,033; p-valor 0,035) e primeira dose de sarampo, caxumba e rubéola (Kappa 0,056; p-valor 0,018) (Tabela 1). 

**Tabela 1:** Número de crianças com dose registrada na caderneta de vacinação e no Sistema de Informações do Programa Nacional de Imunizações e concordância de Kappa entre as duas fontes de informação. Cubatão, 2023 (n=407)

Imunobiológicos	Caderneta de vacinação (n=407)	Sem registro no sistema de informação n=311 (%)	Coeficiente Kappa	p-valor
BCG	404	167 (53,7)	0,011	0,502
Hepatite B	404	188 (60,8)	-0,002	1,000
Vacina inativada da poliomielite – 1^a^ dose	406	102 (32,8)	-0,006	1,000
Vacina inativada da poliomielite – 2^a^ dose	406	134 (43,1)	0,008	0,434
Vacina inativada da poliomielite – 3^a^ dose	402	151 (48,6)	0,026	0,061
Pentavalente – 1^a^ dose	406	102 (33,1)	-0,006	1,000
Pentavalente – 2^a^ dose	406	141 (45,3)	0,008	0,457
Pentavalente – 3^a^ dose	400	161 (51,8)	0,028	0,063
Rotavírus humano – 1^a^ dose	394	101 (32,5)	0,113	<0,001
Rotavírus humano – 2^a^ dose	385	135 (43,4)	0,114	<0,001
Pneumocócica – 1^a^ dose	406	92 (29,9)	-0,006	1,000
Pneumocócica – 2^a^ dose	406	128 (41,2)	0,009	0,415
Pneumocócica – reforço	398	172 (55,6)	0,023	0,245
Meningocócica – 1^a^ dose	406	114 (37,0)	-0,006	1,000
Meningocócica – 2^a^ dose	402	131 (42,1)	0,033	0,035
Meningocócica – reforço	398	183 (59,2)	0,019	0,252
Febre amarela	386	163 (54,0)	0,024	0,316
Sarampo, caxumba e rubéola – 1^a^ dose	399	102 (32,5)	0,056	0,018
Sarampo, caxumba e rubéola – 2^a^ dose	391	170 (55,6)	0,024	0,249
Varicela	391	168 (54,7)	0,035	0,078
Hepatite A	391	162 (52,7)	0,039	0,072
Difteria, tétano e coqueluche – 1º reforço	393	159 (51,8)	0,037	0,070
Poliomielite – 1º reforço	397	164 (53,4)	0,019	0,300

Entre os casos com registro na caderneta e no sistema de informação, avaliou-se a concordância de datas de aplicação das vacinas. Febre amarela e primeiras doses de reforço de poliomielite e de difteria, tétano e coqueluche apresentaram grau de concordância entre 0,60 e 0,79 (concordância substancial). Todas as demais apresentaram concordância acima de 0,80 (concordância quase perfeita). Chama atenção que vacinas que devem ser aplicadas na mesma data apresentaram proporção de concordância diferente, por exemplo, as vacinas BCG (90,2%) e hepatite B (92,6%), as doses de primeira reforço da difteria, tétano e coqueluche (72,3%) e poliomielite (77,7%) e a segunda dose da sarampo, caxumba e rubéola (72,1%) e varicela (90,0%). A diferença de registro de datas de aplicação no sistema de informação variou de 1 dia (hepatite A) a -1.186 dias (febre amarela) em relação à caderneta de vacinação (Tabela 2).

**Tabela 2:** Número de crianças com registro na caderneta de vacinação e no Sistema de Informações do Programa Nacional de Imunizações, número de crianças com registro de datas de aplicação concordantes nas duas fontes de dados, coeficiente de correlação intraclasse (*intraclass correlation coefficient* – ICC) e diferença em dias dos casos com datas discordantes por imunobiológico. Cubatão, 2023 (n=311)

	Número de casos				Valores mínimo e máximo quando há discordância entre as datas (dias)	
Imunobiológico	Com registro da vacina no Sistema de Informações	Com data de aplicação concordante n (%)	ICC	p-valor do ICC	Mínimo	Máximo
BCG	142	128 (90,2)	0,907	<0,001	-1095	335
Hepatite B	121	112 (92,6)	0,997	<0,001	-78	120
Vacina inativada da poliomielite – 1^a^ dose	209	191 (91,4)	0,998	<0,001	-98	3
Vacina inativada da poliomielite – 2^a^ dose	176	152 (86,4)	0,925	<0,001	-833	365
Vacina inativada da poliomielite – 3^a^ dose	156	138 (88,4)	0,950	<0,001	-850	188
Pentavalente – 1^a^ dose	208	191 (91,8)	0,998	<0,001	-112	2
Pentavalente – 2^a^ dose	169	146 (86,4)	0,930	<0,001	-833	61
Pentavalente – 3^a^ dose	145	126 (86,9)	0,944	<0,001	-850	188
Rotavírus humano – 1^a^ dose	200	186 (93,0)	0,989	<0,001	-77	366
Rotavírus humano – 2^a^ dose	159	143 (89,9)	0,932	<0,001	-833	366
Pneumocócica – 1^a^ dose	218	198 (90,8)	0,963	<0,001	-440	31
Pneumocócica – 2^a^ dose	182	161 (88,5)	0,919	<0,001	-833	132
Pneumocócica – reforço	132	113 (85,6)	0,957	<0,001	-662	366
Meningocócica – 1^a^ dose	196	157 (80,1)	0,926	<0,001	-436	89
Meningocócica – 2^a^ dose	176	138 (78,4)	0,895	<0,001	-792	101
Meningocócica – reforço	121	111 (91,7)	0,970	<0,001	-524	217
Febre amarela	132	113 (85,6)	0,731	<0,001	-1186	1181
Sarampo, caxumba e rubéola – 1^a^ dose	203	110 (54,2)	0,809	<0,001	-693	784
Sarampo, caxumba e rubéola – 2^a^ dose	129	93 (72,1)	0,870	<0,001	-512	731
Varicela	130	117 (90,0)	0,875	<0,001	-1044	425
Hepatite A	137	130 (94,9)	0,989	<0,001	-365	1
Difteria, tétano e coqueluche – 1º reforço	141	102 (72,3)	0,770	<0,001	-961	716
Poliomielite – 1º reforço	139	108 (77,7)	0,710	<0,001	-1044	478

## Discussão

Este estudo revelou discrepâncias entre os registros de doses de vacinação nas cadernetas e os dados do Sistema de Informações do Programa Nacional de Imunizações. Embora os resultados mostrem, por meio das cadernetas de vacinação, que Cubatão atingiu as metas de cobertura vacinal para crianças menores de 2 anos nascidas em 2019 e 2020, a baixa completude e precisão dos dados no Sistema de Informações do Programa Nacional de Imunização destacam desafios no registro e na gestão da informação. Tais discrepâncias podem comprometer o monitoramento das coberturas vacinais, afetando a capacidade de avaliar a efetividade das estratégias de imunização. Esses achados reforçam a necessidade de aprimorar a qualidade dos registros para garantir que os esforços de vacinação sejam adequadamente documentados e avaliados.

Registros de vacinação de qualidade são fundamentais para o monitoramento e a avaliação das coberturas vacinais e dos programas de imunização (21). Um sistema de informação efetivo para o monitoramento da cobertura vacinal nos diferentes níveis da assistência à saúde deve incluir alguns componentes essenciais: acesso confiável à internet nas salas de vacinação, interoperabilidade com outras bases de dados, como o registro de nascidos vivos, recursos humanos em número suficiente e bem treinados, financiamento adequado, procedimentos para lidar com registros duplicados e identificação única no sistema, além de procedimentos para evitar a perda de dados e garantir retroalimentação ágil entre os níveis nacional e local (22). Para serem considerados de qualidade, os registros devem ser precisos, completos, relevantes e oportunos (23). A baixa qualidade nos registros de vacinação tem se apresentado como um obstáculo para países de média e baixa renda (24,25). 

São múltiplos os desafios para a implementação de um sistema de registro de vacinação nominal nacional em um país de dimensões continentais e com grande desigualdade regional e socioeconômica, como o Brasil. Entre os desafios, estão a falta de equipamentos, internet contínua e profissionais treinados nos serviços localizados em áreas mais remotas. Pode-se considerar também um obstáculo a existência prévia de sistemas de registro desenvolvidos por municípios com mais recursos, que não querem deixar de utilizar seu próprio sistema. 

A perda de informação durante o processo de envio dos dados dos municípios para a base nacional do Sistema de Informações do Programa Nacional de Imunizações é relatada como mais um desafio para o sistema de informação. Comparando-se os registros de difteria, tétano e coqueluche das salas de vacina de um município em Minas Gerais, com os transferidos para banco de dados nacional do Programa Nacional de Imunizações, foi encontrada concordância em apenas 1 dos 60 (1,6%) meses analisados (8).

A migração do sistema de registro de informações em 2019, que passou a registrar as doses aplicadas nas unidades de saúde no e-SUS Atenção Básica antes da completa implementação do Sistema de Informações do Programa Nacional de Imunizações (3), trouxe dificuldades relacionadas à complexidade da implementação do novo sistema nas salas de vacina e à garantia de que as doses fossem corretamente registradas e transferidas para o nível central sem perdas.

O e-SUS Atenção Básica é um sistema que tem o objetivo de simplificar a coleta, a inserção, a gestão e o uso da informação no nível da Atenção Básica exercendo função vital na rede de informação do SUS (26). O sistema é composto por dois softwares, conforme descritos a seguir.

1. Coleta de Dados Simplificada: sistema de transição/contingência que apoia o processo de coleta de dados por meio de fichas para o registro de informações das ações realizadas pelas equipes de Atenção Básica. É indicado para estabelecimentos de saúde que não possuem conexão de internet e computadores suficientes para os profissionais.
2. Prontuário Eletrônico do Cidadão: sistema com prontuário eletrônico que tem como principal objetivo apoiar o processo de informatização das unidades básicas. É usado para inserir os registros clínicos dos atendimentos e as fichas do sistema Coleta de Dados Simplificada, preenchidas pela equipe em seu processo de trabalho (27).

Em Cubatão, embora as unidades de saúde contassem com computadores e conexão com a internet, o sistema de informação utilizado pela Atenção Básica até início de 2024 era e-SUS Atenção Básica – Coleta de Dados Simplificada que possibilita a digitação individual das doses aplicadas de vacina. Diferentemente do Prontuário Eletrônico do Cidadão, o campo de identificação com dados do Cartão Nacional de Saúde ou do Cadastro de Pessoas Físicas não é de registro obrigatório (28), e a ausência desses dados impossibilita a interoperabilidade entre os sistemas. 

Avaliação da Secretaria Municipal de Saúde de Cubatão, realizada entre janeiro e junho de 2023, identificou que o número de dados registrados manualmente nas salas de vacinação eram 23,4% maiores que os observados nos relatórios do e-SUS Atenção Básica municipal e 58,0% maiores que os dados do Sistema de Informações do Programa Nacional de Imunizações (dados não publicados). 

O Ministério da Saúde tem concentrado esforços na recuperação de dados por meio da identificação de doses represadas que não chegam à Rede Nacional de Dados em Saúde. Em 2023, 91,0% das doses represadas no sistema de informação da Atenção Básica foram atribuídas ao erro “paciente não encontrado” tornando-as inelegíveis para envio imediato à Rede Nacional de Dados em Saúde. Entre essas doses, 99,6% estavam represadas devido ao número de identificação (Cartão Nacional de Saúde ou Cadastro de Pessoa Física), que estavam incorretos ou não informados. Visando à correção desse erro, os municípios têm recebido uma planilha detalhando todas as doses represadas por esse motivo, para que sejam feitas as correções necessárias e o reenvio dos dados (28). 

Além da discordância entre os dados do Sistema de Informações do Programa Nacional de Imunizações e os dados registrados na caderneta de vacinação Cubatão, São Paulo, este estudo revelou que 23,6% das crianças incluídas no inquérito vacinal desse município não possuíam qualquer registro no sistema de informação e em 10% dos casos havia também discordância nas datas de aplicação das vacinas. 

Entre 4.050 crianças com esquema vacinal completo aos 24 meses, 11,0% não tinham registro no Sistema de Informação do Programa Nacional de Imunizações com discordância total entre doses e datas nas duas fontes de 14,0% (18). A maior porcentagem de ausência de registro neste estudo pode ser devido à metodologia. Para avaliar a confiabilidade do sistema de informação, utilizou-se a subamostra de crianças incluídas no inquérito que possuíam esquema completo de vacinação. Neste estudo, a totalidade da amostra independente do status vacinal foi avaliada. Em uma província da China, foi revelado que a proporção de crianças com caderneta de vacinação e sem registro no sistema de informação foi 12,6%, também menor que a deste estudo (29). 

Uma das possíveis explicações para essas discordâncias é a prática de não realizar a digitação diretamente no sistema no momento da vacinação. O uso do papel como meio de registro e consequentemente a não digitação em tempo real podem gerar sub-registro e comprometer a qualidade dos dados. Fatores como a baixa conectividade com a internet, a lentidão do sistema e o fato de os dados vacinais às vezes serem digitados por outro profissional são apontados como impedimentos para a digitação em tempo real (13,28,30,31). Outro fator que pode ter contribuído para a ausência de registro nesse período foram as condições de trabalho impostas pela pandemia de covid-19. Devido ao aumento de casos de adoecimento entre os profissionais e ao redimensionamento das equipes em hospitais e unidades de pronto atendimento, exigiu-se a realocação de trabalhadores para os setores de linha de frente no atendimento aos casos de covid-19, sobrecarregando outras áreas (32).

Uma questão importante para melhoria dos dados em nível municipal é a implementação da “cultura de utilização de dados”. Sabe-se que, sem o uso permanente das informações geradas, não haverá incentivo para a melhoria da coleta (33). Dados de baixa qualidade não serão utilizados e, justamente por não serem usados, permanecerão de baixa qualidade. O uso ampliado dos dados contribui para a melhoria da coleta e de sua qualidade, o que promove maior uso dos dados (34). 

Uma questão importante para melhoria dos dados em nível municipal é a implementação da “cultura de utilização de dados”. Sabe-se que, sem o uso permanente das informações geradas, não haverá incentivo para a melhoria da coleta (33). Dados de baixa qualidade não serão utilizados e, justamente por não serem usados, permanecerão de baixa qualidade. O uso ampliado dos dados contribui para a melhoria da coleta e de sua qualidade, o que promove maior uso dos dados (34). 

Capacitar e estimular os municípios para utilização dos dados para tomada de decisão pode ser uma estratégia para monitoramento constante e identificação precoce de falhas na coleta de dados. Supervisões às salas de vacinação e feedbacks realizados por níveis centrais também têm sido apontados como estratégias para melhoria da qualidade (35), trazendo um elemento de responsabilidade compartilhada em relação à qualidade dos dados (34).

Experiências internacionais apontam os telefones celulares, já amplamente utilizados também no Brasil, como ferramenta para tornar mais ágil e oportuno o registro de doses aplicadas por meio de aplicativos, mesmo em contextos de baixa conectividade. Exemplos exitosos em países como Moçambique, China e Tailândia reforçam a viabilidade dessas soluções em cenários com recursos limitados (36).

A implementação de tecnologia como a de um sistema de informação da imunização envolve mudanças estruturais e processuais no cotidiano dos profissionais de saúde, configurando-se como um processo complexo (30). Os resultados deste estudo destacam desafios no registro de vacinação em Cubatão, especialmente em relação à precisão e à completude dos dados no Sistema de Informações do Programa Nacional de Imunizações. As discrepâncias entre os dados das cadernetas e o sistema de informação podem ser atribuídas, em parte, à falta de digitação em tempo real e à ausência de registro correto de identificação dos pacientes comprometendo a interoperabilidade dos sistemas e-SUS e Sistema de Informações do Programa Nacional de Imunizações. 

É fundamental a intensificação dos esforços para capacitar as equipes de saúde, aprimorar a infraestrutura tecnológica e assegurar o registro preciso e oportuno dos dados de vacinação. A recuperação de doses represadas e a correção de registros incorretos são essenciais para melhorar a qualidade das informações e, consequentemente, a eficiência dos programas de imunização. Futuros estudos devem continuar a monitorar a cobertura vacinal e avaliar o impacto das intervenções realizadas, garantindo que as metas de vacinação sejam atingidas e refletidas com precisão nos registros oficiais.

## Data Availability

Seguindo diretrizes éticas e legais, e para assegurar a proteção da privacidade dos participantes e o cumprimento das normas do comitê de ética responsável, os dados gerados nesta pesquisa estarão disponíveis mediante solicitação.
